# Primary Ciliary Signaling in the Skin—Contribution to Wound Healing and Scarring

**DOI:** 10.3389/fcell.2020.578384

**Published:** 2020-11-13

**Authors:** Mayu Hosio, Viljar Jaks, Heli Lagus, Jyrki Vuola, Rei Ogawa, Esko Kankuri

**Affiliations:** ^1^Faculty of Medicine, Department of Pharmacology, University of Helsinki, Helsinki, Finland; ^2^Institute of Molecular and Cell Biology, University of Tartu, Tartu, Estonia; ^3^Dermatology Clinic, Tartu University Hospital, Tartu, Estonia; ^4^Department of Plastic Surgery and Wound Healing Centre, Helsinki University Hospital, University of Helsinki, Helsinki, Finland; ^5^Helsinki University Hospital, University of Helsinki, Helsinki, Finland; ^6^Department of Plastic, Reconstructive and Aesthetic Surgery, Nippon Medical School, Tokyo, Japan

**Keywords:** primary cilia, cell signaling, scar formation, wound healing, inflammation, fibroblast, myofibroblast

## Abstract

Primary cilia (PC) are solitary, post-mitotic, microtubule-based, and membrane-covered protrusions that are found on almost every mammalian cell. PC are specialized cellular sensory organelles that transmit environmental information to the cell. Signaling through PC is involved in the regulation of a variety of cellular processes, including proliferation, differentiation, and migration. Conversely, defective, or abnormal PC signaling can contribute to the development of various pathological conditions. Our knowledge of the role of PC in organ development and function is largely based on ciliopathies, a family of genetic disorders with mutations affecting the structure and function of PC. In this review, we focus on the role of PC in their major signaling pathways active in skin cells, and their contribution to wound healing and scarring. To provide comprehensive insights into the current understanding of PC functions, we have collected data available in the literature, including evidence across cell types, tissues, and animal species. We conclude that PC are underappreciated subcellular organelles that significantly contribute to both physiological and pathological processes of the skin development and wound healing. Thus, PC assembly and disassembly and PC signaling may serve as attractive targets for antifibrotic and antiscarring therapies.

## 1. Introduction

The wound healing cascade progresses through the partially overlapping stages of hemostasis, inflammation, repair/proliferation, and remodeling (Almine et al., 2012). Failure of the cascade to progress through these stages gradually and in due order delays wound healing and can lead to excessive scarring or the formation of chronic wounds and ulcers (Landén et al., 2016). In the hemostasis phase, activated platelets induce formation of a fibrin clot and trigger the release of growth factors, such as platelet-derived growth factor (PDGF) and transforming growth factor β-1 (TGFβ-1). Production of growth factors, cytokines, and chemokines throughout the inflammatory stage results in coordinated activation of the immune system (Liu et al., 2003). The inflammatory phase is characterized by the migration of leukocytes into the injured skin. The coordinated activities of these cells help in clearing microbes, foreign material, and cell debris from the wound, setting the stage for the proliferation phase.

During the wound repair/proliferation phase, fibroblasts, and endothelial cells of blood vessels in the wound bed start to proliferate and form a highly vascularized fibrotic tissue that is rich in extracellular matrix (ECM) components to fill the tissue defect. Also, epithelial cells within or surrounding the wound proliferate and begin to migrate to re-surface the wound.

Due to the uneven, granulated appearance of the surface, it is called granulation tissue. It is a mixture of vascular structures, fibroblasts, macrophages, collagen bundles, fibronectin, and hyaluronic acid. Granulation tissue can be found also in chronic wounds with delayed epithelialization (Martin and Nunan, 2015). In the repair/proliferation phase, the formation of granulation tissue improves the structure and function of the wounded skin (Schultz and Wysocki, 2009). Granulation tissue cells produce TGF-β 1 and TGF-β 2, which in turn induce fibroblasts to proliferate and synthesize the provisional ECM. In hypertrophic scars, this fibroblast response is left unchecked, causing excessive cell proliferation and ECM production (Penn et al., 2012).

Upon the transition from granulation tissue to a scar, in the remodeling phase, the abundant type III collagen that dominates the proliferation stage ECM is largely replaced by type I collagen through the balanced activity of matrix metalloproteinases and their tissue inhibitors (Singer and Clark, 1999). This leads to the formation of a relatively acellular scar. Disturbances in the balance between collagen synthesis and degradation also impair normal healing and can contribute to the development of pathological scars (Sussman and Bates-Jensen, 2007). Moreover, the inflammatory signals in the forming scar are sustained, the scar tissue continues to grow forming hypertrophic scars and keloids. While a hypertrophic scar extends outward from the skin surface and does not surpass the limits of the original tissue defect, the keloid tissue spreads into the wound edges (Ogawa, 2017).

The aim of this review is to summarize the current understanding of primary cilia (PC) functions and their main signaling pathways associated with inflammation, scar formation, and wound healing under normal and pathological conditions. We collected literature across species, cell, and tissue types as well as disease models to provide further insight into the possible roles of the PC in skin.

## 2. Primary Cilia

### 2.1. History of Primary Cilia

Cell membrane protrusions that carry out essential functions are present in archaea, bacteria, and eukaryotes. The motile hairlike flagella of archaea and bacteria and the motile cilia of eukaryotes share structural similarities (Fisch and Dupuis-Williams, 2011). They are comprised of a 9+2 axoneme, a circular structure consisting of nine microtubule doublets enclosing a central pair of microtubules (Fisch and Dupuis-Williams, 2011). In addition to these motile cilia, the mammalian ciliary subtypes include motile embryonal nodal cilia with a 9+0 axoneme, sensory nonmotile kinocilia with a 9+2 axoneme and PC (Satir and Christensen, 2008). PC are singular and nonmotile, and have a 9+0 axoneme thus lacking central microtubule pair.

The primary cilium was first discovered in the renal epithelium and thyroid gland in 1898 by Zimmerman, who named it “Zentralgeissel” or central flagellum (Zimmermann, 1898). In 1962, the name “primary cilium” was introduced by Sorokin (1962). They described PC in the central nervous system (Sorokin, 1962), and later on fibroblasts and smooth muscle (SM) cells (Sorokin, 1968).

The sensory functions of PC (Pedersen et al., 2006; Singla and Reiter, 2006; Berbari et al., 2009) were initially already suggested by Zimmermann (1898). However, until their association with human disorders in the twenty-first century, PC were largely neglected. Ciliopathies, which are syndromes with genetic alterations in genes coding for PC-associated proteins, include polycystic kidney disease, Bardet-Biedl syndrome (BBS), Joubert syndrome, Meckel Gruber syndrome, Ellis-van Creveld syndrome, and Jaune syndrome. More attention was given to PC after Huangfu et al. (2003) associated them with the Hedgehog (Hh) pathway that regulates cell proliferation and differentiation and has implications in cancer (Gupta et al., 2010; Sari et al., 2018). The cell-membrane-covered axoneme is comprised of nine microtubule doublets (9+0 axoneme) that extend from the basal body (Ishikawa and Marshall, 2011) and protrude into the extracellular space (Adams, 2010; Hoey et al., 2012).

### 2.2. Structure of Primary Cilia

A primary cilium consists of three parts: (1) the basal body, (2) the transition zone, and (3) the axoneme (Ishikawa and Marshall, 2011; Seeger-Nukpezah and Golemis, 2012) (**Figure 2**).

**Figure 1:**
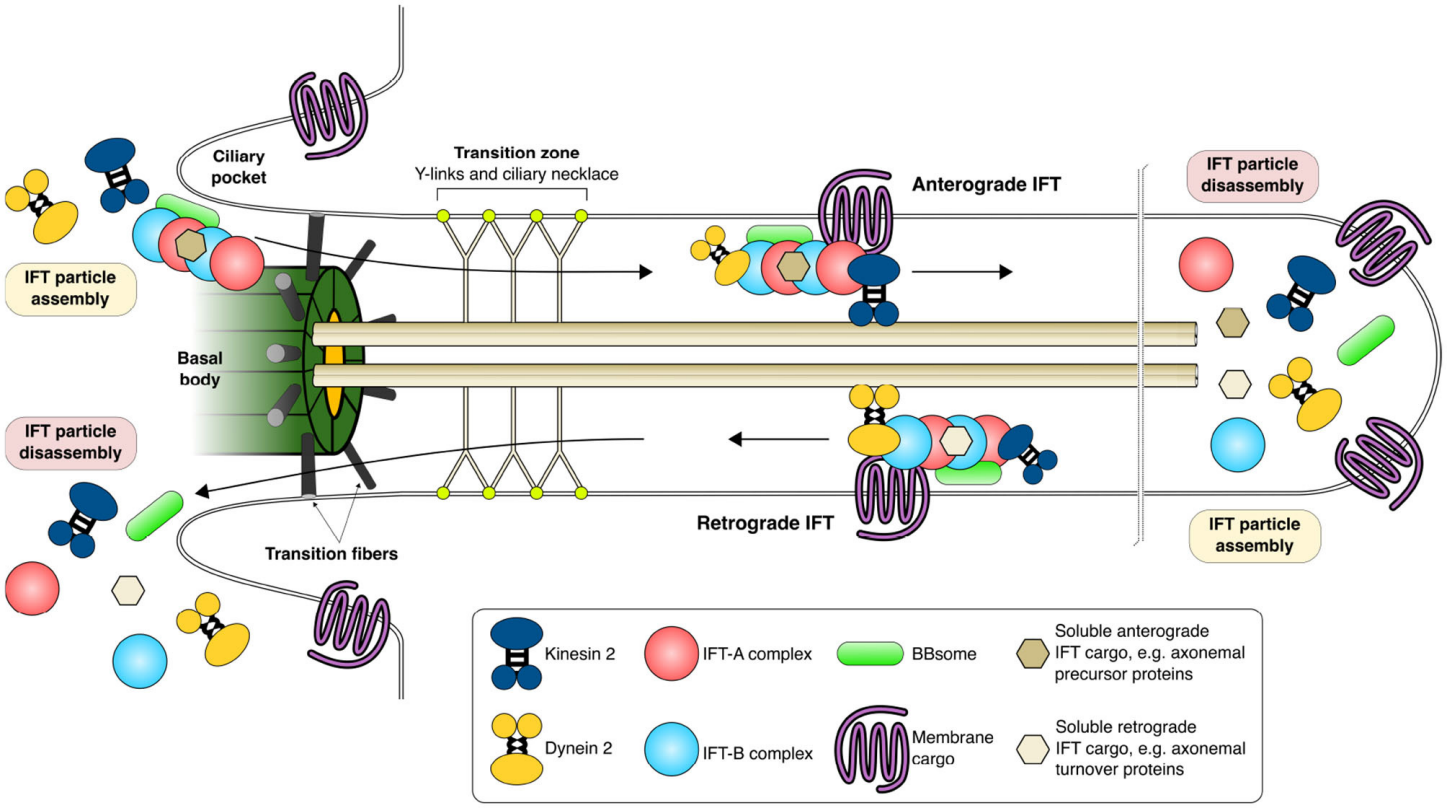
Intraflagellar transport and functional maintenance of the primary cilium. IFT particles are assembled in the cytoplasm and are transported through the transition zone coupled to IFT trains, multi-megadalton complexes composed of IFT-A and -B proteins, kinesin, dynein, BBsome proteins as well as soluble and membrane cargoes. Anterograde IFT carries cargo toward the tip of the primary cilia whereas retrograde IFT carries cargo away from the primary cilia toward the cytoplasm. Kinesins are motor proteins that move along the axoneme in anterograde IFT while dyneins participate in retrograde IFT. The primary cilia is formed from a modified mother centriole (basal body) from which the nine pairs of axonemal tubules grow and extend out from the cell surface covered by the plasma membrane. The plasma membrane composition of the primary cilia is specifically maintained and guarded by the transition fibers and transition zone (Y-links and ciliary necklace) through which only specific proteins and their cargo are allowed. References for this figure (Cole and Snell, 2009; Basten and Giles, 2013; Nachury, 2014; Taschner and Lorentzen, 2016; Eguether and Hahne, 2018; Wingfield et al., 2018).

The membrane of the primary cilium is enriched in specific signaling molecules, such as transmembrane receptors and phosphoinositides that are essential for chemical sensing and signaling (Anvarian et al., 2019). The transition zone is located between the basal body and the axoneme and contains protein complexes that, together with gatekeeper structures such as Y-links and basal body transition fibers, control the incoming and outgoing stream of ciliary proteins (Garcia-Gonzalo and Reiter, 2017). The base of the cilium is flanked by membrane invaginations called ciliary pockets that are important for the internalization of specific signaling molecules. Figure 1 shows a crosscut section of a PC with its subsections together with proteins involved in PC maintenance.

### 2.3. Assembly and Disassembly of Primary Cilia

**Figure 2:**
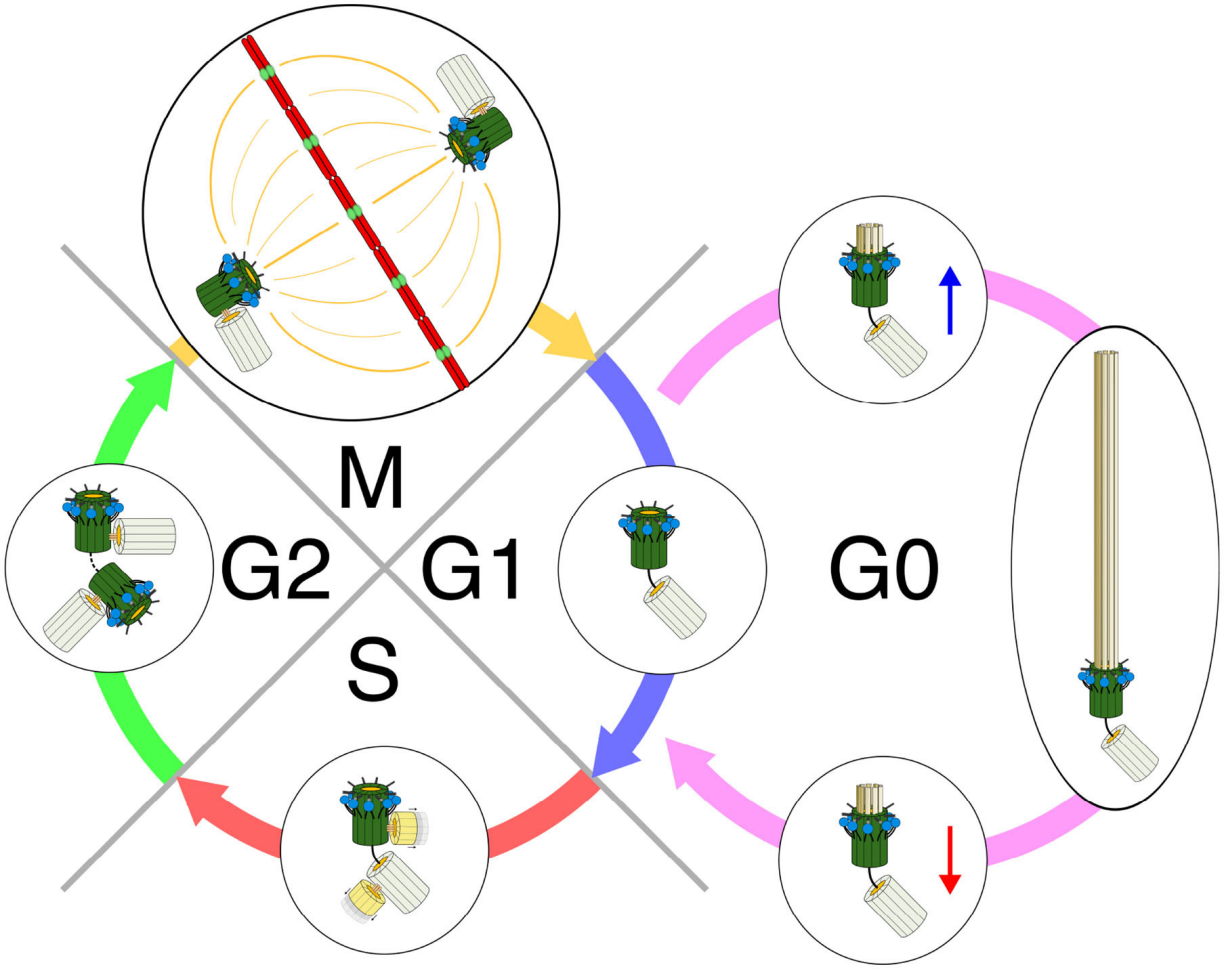
Primary cilia, centrioles, and cell cycle. Early G1—Centrioles are disengaged from their orthogonal orientation. The mother centriole with its distal appendages and its accompanying daughter centriole with its pericentriolar protein-rich matrix form the basal body for ciliation. G0—Basal body (BB) migrates to the plasma membrane. Plasma membrane invaginates and centriolar elongation progresses in ciliary vesicles. The BB anchors to and fuses with the cell membrane. Axonemal growth is initiated. A functional primary cilia is formed. Its length and function are dynamically controlled by constant turnover as well as both cellular and environmental cues. Late G1—Before re-entry to the cell cycle, the first wave of primary cilia disassembly occurs. Ciliary shortening is required to allow the cell to enter the S-phase. S—Both mother and daughter centrioles duplicate to form new mother-daughter pairs. Duplication occurs in an orthogonal orientation from the existing centrioles. The newly formed centrioles become the new daughter centrioles, with a new mother centriole established from the old daughter for its respective cell after division. G2—The second wave of cilia disassembly and resorption occurs and is finalized in order for the cell to enter mitosis. The new mother centriole (old daughter) acquires its distal and sub-distal appendages. M—Centrioles in their engaged, orthogonal orientation form the centrosomes for mitosis and subsequent cell division. Redrawn using information from the following: Ishikawa and Marshall (2011), Basten and Giles (2013), and Wang and Dynlacht (2018).

Ciliogenesis is tightly orchestrated by cell cycle progression (Prescott, 1970; Sánchez and Dynlacht, 2016) (Figure 2). The assembly of a primary cilium starts after the end of mitosis (Sánchez and Dynlacht, 2016). Small cytoplasmic vesicles (preciliary vesicles) originating from the Golgi complex and endosomal recycling compartment are first transported by specific kinesins to the distal end of the mother centriole to form a larger ciliary vesicle (Sorokin, 1962; Schmidt et al., 2012; Kobayashi et al., 2014; Lu et al., 2015b). From within this vesicle, the membrane-surrounded axoneme grows to extend out of the cell surface, covered by the plasma membrane (Gilula and Satir, 1972).

As the axonemal structure grows in length, the assembled microtubules are stabilized through acetylation of tubulin by specific tubulin acetyltransferases (α-tubulin K40 acetyltransferase, αTAT, and MEC-17) (Leroux, 2010). It has been suggested that this process is activated by the Aurora-A centrosomal kinase (AURKA) that controls mitotic entry via activation of cyclin-dependent kinase 1 (CDK1) B (Marumoto et al., 2005; Pugacheva et al., 2007). The disassembly of PC by AURKA requires the activity of histone deacetylase 6 (HDAC6). Mirvis et al. (2019) demonstrated that PC disassembly through these molecular signals could occur through at least two mechanisms: through gradual degradation and incorporation of the axoneme into the cell or through whole-cilium shedding (deciliation) with the axoneme being excised at its base.

Altogether, the formation, maintenance, and degradation of the primary cilium are highly regulated processes. The dynamic interrelationship between the assembly and disassembly processes determines the length of the cilium (Keeling et al., 2016). Relative to the length of the cilium, the cell's ability to sensitively detect extracellular cues is either increased or decreased. PC length can affect mechanotransduction, the process by which cells transduce mechanical forces into biological signals (Spasic and Jacobs, 2017) or cause defects in signal processing (Canterini et al., 2017).

### 2.4. Signaling Pathways

PC guide such fundamental cellular functions as proliferation and differentiation and thereby contribute to organ development, tissue homeostasis, and repair as well as the regulation of inflammation (Pazour et al., 2000; Huangfu et al., 2003; Marshall and Nonaka, 2006; Singla and Reiter, 2006; Berbari et al., 2009; Green and Mykytyn, 2014). Signaling through Wnt Gerdes et al. (2007), Notch (Ezratty et al., 2011), Hh (Huangfu et al., 2003), G-protein-coupled receptors (GPCRs) (Schou et al., 2015), TGF-β (Clement et al., 2013), and insulin-like growth factor-1 (IGF-1) (Yeh et al., 2013) has been associated with PC. All these signaling pathways are important for skin development and contribute to wound repair (Bielefeld et al., 2013).

**Figure 3:**
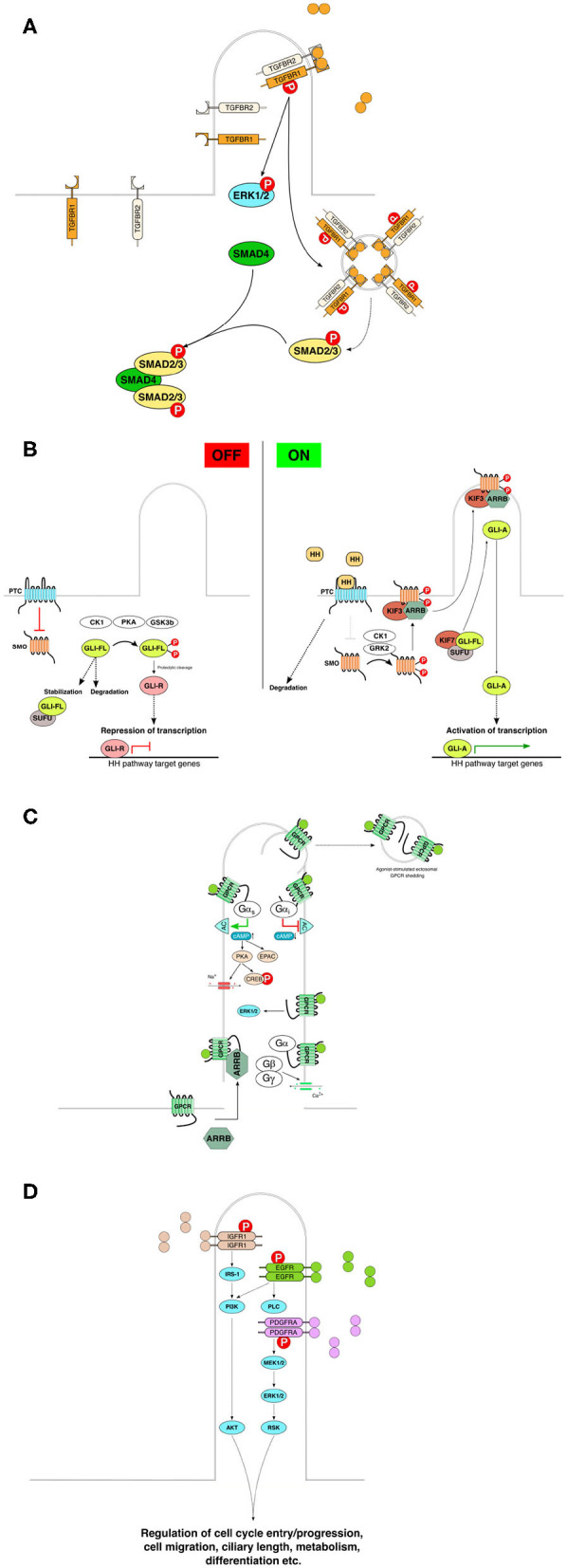
Basic components and rough characteristics of each indicated signaling pathway in relation to primary cilia. **(A)** TGFbeta receptors accumulate in the primary cilia, and ligand binding to the TGFBR2 faciliates recruitment of TGFBR1 to form the heterodimeric receptor complex. Subsequent phosphorylation and activation of the receptor leads to downstream activation of the SMAD family of transcription factors by active receptor transport and aggregation in endosomal vesicles for downstream signal enhancement. **(B)** Mammalian Hedgehog signaling is tightly linked to the primary cilia. In the absence of signal, the pathway is maintained inactive or in a repressive state through the inhibitory action of Patched on Smoothened, and through the proteolytic cleavage or SUFU-mediated stabilization of GLI family of transcription factors. Upon activation by Hedgehog pathway ligands (Sonic Hedgehog, Indian Hedgehog or Desert Hedgehog) the inhibitory activity of PTC on SMO is abrogated and SMO can, with the help of beta-arresin and kinesin 3, translocate to the primary cilia. Active SMO then facilitates dissociation of GLI from its regulator SUFU leading to activation of GLI and GLI-A-induced transcription of HH pathway response genes in the nucleus. **(C)** The primary cilia-associated G-protein-coupled receptors are transported to the primary cilia assisted by e.g., beta-arrestin. Ligand stimulation leads to receptor activation and activation of downstream signaling depending on the receptor's coupling to various types of G-proteins. Ligand-induced shedding of G- proteins in ectosomes from the primary cilia denotes an additional level of regulation or suppression of G-protein signaling. **(D)** Receptor tyrosine kinase pathways are also associated with the primary cilia. For example, IGF, EGF, and PDGF utilize primary cilia for signaling. Ligand binding results in downstream activation of specific kinase cascade activations leading to activation of transcription factors and intracellular effectors that participate in the regulation of various cellular processes. Redrawn using information from the following: Ruel and Thérond (2009), Ishikawa and Marshall (2011), Christensen et al. (2012), Hilgendorf et al. (2016), Bangs and Anderson (2017), Christensen et al. (2017), and Anvarian et al. (2019). BBsome, Bardet-Biedl syndrome protein complex; IFT, intraflagellar transport; PTC, patched; SMO, smoothened; SUFU, suppressor of fused homolog; GLI-FL, glioma-associated oncogene (zinc finger protein GLI) full-length; GLI-A, activated form of GLI; GLI-R, repressor form of GLI; CK1, casein kinase 1; PKA, protein kinase A; GSK3b, glycogen synthase kinase 3 beta; HH, hedgehog; GPRK2, G-protein- coupled receptor kinase 2; ARRB, beta-arrestin; KIF, kinesin superfamily protein; TGFB, transforming growth factor beta; TGFBR, TGFB receptor; SMAD, family of transcription factors homologous to *C. elegans* SMA and MAD families; ERK, extracellular signal-regulated kinase; MEK, mitogen-activated protein kinase kinase; IGFR, insulin-like growth factor receptor; EGFR, epidermal growth factor receptor; PDGFRA, platelet-derived growth factor receptor alpha; IRS, insulin receptor substrate; PI3K, phosphoinositide 3-kinase; PLC, phospholipase C; AKT, protein kinase B; RSK, ribosomal protein S6 kinase; GPCR, G-protein-coupled receptor; AC, adenylate cyclase; cAMP, cyclic adenosine monophosphate; CREB, cAMP-responsive element-binding protein; EPAC, rap guanine nucleotide exchange factor, exchange protein directly activated by cAMP; G alpha s, G-protein alpha subunit, stimulatory; G alpha i, G-protein alpha subunit, inhibitory; Gbeta, gamma, G-protein beta and gamma subunits, respectively; RTK, receptor tyrosine kinase.

The primary cilium acts as a signaling hub that coordinates the activity of numerous signaling pathways (Nishimura et al., 2019), including in addition to the above-mentioned signaling pathways signaling transmitted by and associated with bone morphogenetic protein (BMP) (Clement et al., 2013), planar cell polarity (Ross et al., 2005), platelet-derived growth factor receptorα (PDGFRα) (Schneider et al., 2005), receptor tyrosine kinases (RTKs) (Christensen et al., 2012), Hippo (Yu and Guan, 2013), NFkappaB (Karin, 1999), mTOR (Pala et al., 2017), and ECM receptors (McGlashan et al., 2006). In many cases, the primary cilium integrates simultaneous inputs, thus fine-tuning and integrating signals from different sources into an orchestrated cellular response. Examples of signaling cascades associated with PC are presented in Figure 3.

#### 2.4.1. TGF-β

TGF-β/BMP signaling plays a crucial role in cell proliferation, migration, differentiation, apoptosis, ECM remodeling, immune functions, and tumor metastasis (Guo and Wang, 2009), and is one of the major signaling pathways associated with myofibroblast differentiation and epithelial-mesenchymal transformation (Thannickal et al., 2003).

Of the three TGFβ isoforms, TGFβ-1 is the main signaling molecule in most tissue types and pathological processes, including skin and cutaneous wound healing (Wang, 2001; Barrientos et al., 2008). In the skin, TGFβ-1 is expressed in the stratum granulosum and stratum corneum, while TGFβ-2 and -3 are expressed in the supra-basal layers, suggesting that each TGF-β isoform has a different function in keratinocyte proliferation and differentiation (Gold et al., 2000; Cho et al., 2004). While TGF-β-1 and -2 promote scar tissue formation, TGF-β-3 reduces scar formation (Lin et al., 1995; Shah et al., 1995). However, the TGF-β-1 and -2 receptors are present both in fetal and adult dermal tissues (Helmo et al., 2013). Soo et al. (2003) suggested that increased levels of TGF-β-3 expressed early in fetal wounds may compete with TGF-β-1 and -2 to bind to the type II receptor and, moreover, that an anti-scar effect of TGF-β-3 is seen after the early TGF-β-3 induction in fetal wounds or after early application to adult wounds.

There is an increasing body of evidence that PC play an important role in both canonical and non-canonical TGF-β/BMP signaling and, more importantly, in fine-tuning the balance of these pathways (Anvarian et al., 2019) (Figure 3A). It has been shown that in an inactive state, the TGF-β receptors accumulate at the tip of the primary cilium (Clement et al., 2013).

TGF-β/BMP signaling is induced via activation of heterotetrameric type I (RI) and type II (RII) receptor complexes that act as serine/threonine kinases. Upon ligand binding, the receptors are translocated to the base of the cilium and are internalized via clathrin-dependent endocytosis. The activation of TGF-β receptors leads to phosphorylation and activation of transcription factors, small mothers against decapentaplegic (SMAD) 2/3 (Huang and Chen, 2012; Clement et al., 2013). Activated SMAD2/3 bind to and induce the nuclear translocation of a related molecule SMAD4 and the formation of a transcriptionally active complex with SMAD4 regulating thereby gene expression (Clement et al., 2013). Also, clathrin-independent extracellular regulated kinase 1/2 (ERK1/2) activation by TGF-β receptors is located at the ciliary base (Clement et al., 2013). The exact molecules that are involved in the trafficking of TGF-β receptors along primary cilium are not yet described. Nevertheless, the trafficking of Ras-related protein Rab-11A (RAB11), which is involved in endosomal recycling of TGF-β receptors is impaired by the loss of the mother centriole protein centrosomal protein of 128 kDa (CEP128) that coordinates the localization of GF-β receptors, resulting in impairment of TGF-β signaling (Mitchell et al., 2004; Westlake et al., 2011; Mönnich et al., 2018). Non-canonical TGF-β/BMP signaling involves, for example, activation of extracellular signal-regulated protein kinase (ERK)1/2, which in turn activates MAP kinase (Clement et al., 2013).

Interestingly, the negative feedback regulator of TGF-β signaling, SMAD7, and the E3 ubiquitin-protein ligase SMURF1 also localize to the base of the primary cilium and have been suggested to thereby limit excessive TGF-β/BMP signaling (Clement et al., 2013; Heldin and Moustakas, 2016; Miyazawa and Miyazono, 2017; Koefoed et al., 2018).

#### 2.4.2. Wnt/Catenin

The wnt-PCP pathway has been implicated in the regulation of cell morphology, migration, and oriented cell division and has been shown to involve Ryk, Ror family kinases, and the Vangl protein (Green et al., 2014; Yang and Mlodzik, 2015). Many of the core components of the Wnt pathway have been found in PC, and several proteins implicated in different stages of the Wnt signaling cascade have been found to localize to the base of PC (Corbit et al., 2008; Chen et al., 2011; Lancaster et al., 2011).

There are three major types of Wnt signaling: the β-catenin pathway, which is considered the canonical Wnt pathway, and the non-canonical planar cell polarity (PCP) and Wnt/Ca2+ pathways (MacDonald et al., 2007; Semenov et al., 2007; Houschyar et al., 2015). Wnt signaling or the Wnt/β-catenin pathway plays an essential role in skin development and maintenance (Logan and Nusse, 2004; Fuchs, 2007; Clevers and Nusse, 2012). It has also been closely associated with tissue regeneration and repair. The ligands of this pathway include a number of vertebrate homologs of the Drosophila wingless (wnt) gene that bind to the frizzled family of receptors and numerous coreceptors (MacDonald and He, 2012; Niehrs, 2012). Binding to the Frizzled (Fzd) receptor, which leads to complex formation with the coreceptors LRP5/6 and Disheveled (DVL). As a result, β-catenin is stabilized and translocates to the nucleus to regulate gene expression (Kim et al., 2013; Sineva and Pospelov, 2014).

There are conflicting data regarding the role of ciliary transport in the regulation of Wnt signaling. On the one hand, it has been shown that the primary cilium acts as an inhibitor of Wnt signaling, as the loss of the key molecular motors Kif3A, intraflagellar transport 88 (IFT88), and Ofd1 was associated with augmented signaling in response to Wnt3A (Corbit et al., 2008). On the other hand, it was shown that Kif3A might inhibit Wnt signaling independent of the primary cilium (Kim et al., 2016), leaving much to be investigated in future studies.

#### 2.4.3. Notch

The Notch signaling pathway regulates the development and homeostasis of many types of tissues, such as the nervous system, the vascular system, the hematopoietic system, somites, the muscle, the skin, and the pancreas (Hansson et al., 2004). The Notch signaling pathway controls epidermal differentiation (Watt et al., 2008), and it has also been suggested that the Notch pathway is associated with wound repair (Thélu et al., 2002; Chigurupati et al., 2007; Outtz et al., 2010).

In mammals, there are four different Notch receptors that are activated by ligands belonging to the family of Delta-like and Jagged proteins (Brittan and Wright, 2007). Ligand binding induces cleavage of the intracellular domain, which enters the nucleus and activates transcription of target genes (Moore and Alexandre, 2020). A small amount of evidence has indicated that loss of the key transport proteins Kif3a and IFT88 results in disruption of Notch signaling in cultured keratinocytes and the intact epidermis. However, the exact mechanism by which PC regulate Notch signaling is not yet clear (Ezratty et al., 2011).

#### 2.4.4. Hedgehog

The Hh signaling pathway is involved in tissue development, homeostasis, and repair as well as in regulating morphogenesis of the skin during embryogenesis (Ingham and McMahon, 2001; Bielefeld et al., 2013).

There are three Hh proteins in mammals, namely, Sonic hedgehog (Shh), Indian hedgehog (Ihh), and Desert hedgehog (Dhh) (Tasouri and Tucker, 2011) (Figure 3B). Shh plays a major role in skin development and maintenance, while Ihh is involved in cartilage formation and Dhh plays a role in gonadal morphogenesis (Bitgood and McMahon, 1995; Boras-Granic et al., 2006). In addition to its role in normal skin homeostasis, Hh signaling is the main player in the development of the most widespread human malignancy—basal cell carcinoma (Kasper et al., 2012).

The central signaling transducer is the 7-transmembrane molecule Smoothened (Smo), which is under constant inhibition of the 12-transmembrane molecule Patched (Ptch), which acts as a Hh receptor. As a result, the transcriptional regulator GLI3 is processed via protein kinase A-mediated phosphorylation into a repressor form that suppresses the transcription of Hh target genes (Mukhopadhyay and Rohatgi, 2014). Upon ligand binding, Ptch inhibition of Smo is relieved, resulting in the accumulation of transcriptionally active forms of GLI2 and subsequent activation of target gene expression (Kasper et al., 2012). The exact mechanism of signal transduction from Ptch to Smo is not clear; however, a role for cholesterol or cholesterol derivatives, such as oxysterols, has been suggested (Kinnebrew et al., 2019).

The non-canonical Hh signaling pathway involves the activation of GLI transactivators independently of Smo. Most of the key events of Hh signaling are coordinated by the primary cilium. Ptch is localized in caveolin-1-containing membrane lipid rafts at the base of the primary cilium together with SMURF1 and SMURF2 (Yue et al., 2014). Caveolin-1 accumulation at the base of the cilium is dependent on the molecular motor protein KIF13B, and both of these proteins have a considerable impact on Hh signaling (Schou et al., 2017). Upon Hh ligand binding, Ptch1 is cleared from the membrane due to its ubiquitination; consequently, Smo molecules are transported into the ciliary membrane from other parts of the cell (Corbit et al., 2005; Rohatgi et al., 2007).

It has been demonstrated that interaction between Smo and a ciliary protein, Evc, that is localized close to the transition zone is crucial for Hh signal activation (Dorn et al., 2012). As a result, GLI2 and GLI3 are transported by an the IFT protein Tg737 to the tip of the cilium. Loss of this protein alters the correct processing of both active and repressive forms of GLI1 and GLI3, suggesting that the processing events themselves may take place in the tips of the cilia (Haycraft et al., 2005). KIF7, an atypical kinesin, is also involved in the transport and correct processing of the GLI proteins at the tips of cilia, potentially via modulating by modulating the ciliary architecture (Liem et al., 2009; He et al., 2014). Another ciliary protein, GPR161, that belongs to the GRCRs, acts as a repressor for of Hh signaling by promoting the processing of GLI3 into the repressive form, most likely by activation of PKA via cAMP upregulation. Interestingly, the ciliary localization of GPR161 is dependent on IFT-A complex components, most notably Tulp3, which is an adaptor protein that is responsible for the transport of specific GPCRs into the cilium (Mukhopadhyay et al., 2013).

It appears that PC retrograde trafficking of Hh pathway complexes is important for proper Hh signaling, as disruption of IFT 25 and 27 results in the accumulation of Ptch-1 and Smo in the primary cilium and the disruption of Hh signaling (Keady et al., 2012; Eguether et al., 2014). The primary cilium may also play a role in non-canonical Hh signaling. It has been shown that the IFT protein IFT80 can repress activation of the GTPase RhoA by Shh, balancing the canonical and non-canonical Hh signaling pathways (Yuan et al., 2016).

#### 2.4.5. G-Protein-Coupled Receptors

The primary cilium functions as a hub for GPCR signaling (Figure 3C). GPCRs, the largest class of proteins, regulate numerous functions in the cells. The GPCR repertoires of various cell types differ. For example, the majority of neurons located in the mammalian brain have cilia that are enriched in specific GPCRs. GPCRs recruit G-protein heterotrimers consisting of Gα, Gβ, and Gγ subunits. Signal transduction through GPCRs involves the replacement of GDP by GTP on the Gα subunit. Gα and Gβ γ subunits dissociate, and the activated Gα subunit stimulates or inhibits adenyl cyclases (ACs) or activates phospholipase-C (PLC).

The plethora of signaling events that are affected by Gβ γ subunits is only beginning to emerge. Canonically Gβ γ signaling affects the activity of not only potassium and calcium ion channels and PLC but also PI3K, MAP kinases, and some AC isoforms. Hydrolysis of bound GTP to GDP reassociates the Gα and Gβ γ subunits and thus ends their signaling activity (Khan et al., 2013; Hilger et al., 2018). Defects in cilia, as seen in the case of ciliopathies, also affect the nervous system (Guemez-Gamboa et al., 2014). Inactivating mutations in several genes encoding ciliary proteins result in defects in GPCR accumulation in cilia. For example, disruption of a gene encoding a BBS complex (BBSome) protein disrupts the ciliary localization of somatostatin receptor subtype 3 (SSTR3), melanin-concentrating hormone receptor 1 and neuropeptide Y (NPY) receptor subtypes 2 and 5 (NPY2R and NPY5R) (Berbari et al., 2008; Loktev and Jackson, 2013; Green et al., 2016).

In the case of SSTR3, its ciliary localization is dependent on the adaptor protein β-arrestin (Green et al., 2016). The lack of NPY2R and NPY5R in mouse hypothalamic neurons results in excessive food intake, as signaling via these receptors is required for sensing the neuropeptide Y signal that controls sufficient food intake (Loktev and Jackson, 2013). Obesity is also a symptom of melanocortin 4 receptor (MC4R) mutations that impair its ciliary localization in both humans and mice, suggesting that ciliary localization is essential for the functionality of this GPCR receptor (Davenport et al., 2007; Siljee et al., 2018).

#### 2.4.6. Receptor Tyrosine Kinases

RTKs form a prominent class of signal transducers that can be subdivided into several subclasses depending on their specific properties (Lemmon and Schlessinger, 2010). RTKs act as receptors for a wide variety of biomolecules. Most RTKs homodimerize upon ligand binding, which induces autophosphorylation and activation of the RTK subunits.

Subsequently, the activated RTK complex phosphorylates downstream targets that include different intracellular kinases (the MAP kinase family, PI3K-AKT pathway kinases, PL Cγ, and others), which exert the effects induced by specific ligand molecules (Crudden et al., 2018). Signaling through PDGFRα, insulin receptor (IR), and IGF-1 receptor (IGF-1R) is tightly connected to PC (Christensen et al., 2012) (Figure 3D).

#### 2.4.7. PDGFRα

PDGFRα signaling is disrupted by inactivation of the IFT88 and IFT172 transporters, which also causes aberrant cilium assembly (Schneider et al., 2010). Furthermore, IFT20, another ciliary protein, stabilizes the CBL E3 ubiquitin ligases that target PDGFRα for degradation and thereby limit PDGF signaling. Inactivation of IFT20 results in ciliary collapse, inactivation of CBL proteins, and excessive uncoordinated signaling from PDGFRα (Schmid et al., 2018). Ciliary IGF-1R signaling is crucial for adipogenesis. While IGF-1R is localized to the ciliary membrane, its primary targets, insulin receptor substrate 1 (IRS-1) and AKT kinase, reside at the base of the cilium, facilitating coordinated signal transduction and activation of adipogenic differentiation upon receptor activation (Zhu et al., 2009).

## 3. Primary Cilia in Skin Cells and Wound Healing

### 3.1. Primary Cilia, Inflammation, and Skin Immune Cells

Neutrophils are the first cells to be recruited from the circulation to the site of injury in the early stage of inflammation (Sinno and Prakash, 2013). As such, neutrophils have a relatively short lifespan, but the survival signals, such as cytokines, chemokines, and growth factors, in the wound environment can prolong their lifespan. Activated neutrophils produce several proinflammatory and proangiogenic mediators, for example, vascular endothelial growth factor (VEGF), TNF-α, and interleukin 1 (IL-1), to recruit more immune cells and enhance the innate response as well as to promote the progression of wound healing to the inflammatory stage. Further, because neutrophils produce reactive oxygen species and antimicrobial peptides that are essential for wound decontamination in the early inflammatory phase, the failure of subsequent neutrophil apoptosis leads to the persistence of these factors in the wound. Such aggravation of the inflammatory phase can provoke delays in wound healing (Landén et al., 2016). Macrophage activity, on the other hand, can counteract these signals and promote neutrophil apoptosis (Meszaros et al., 2000). Circulating monocytes infiltrate the wound and differentiate into macrophages to facilitate wound healing. Macrophages phagocytose and clear apoptotic neutrophils. This process, called efferocytosis, induces macrophages to produce factors that help limit inflammation and aid wound healing to promote progression to the proliferative phase (Koh and DiPietro, 2011). Macrophages and monocytes are pivotal cells in antimicrobial immune defense and the repair of tissue damage repair. The absence of macrophages leads to impaired wound healing (Mirza et al., 2009), and it has been shown that macrophages play important roles during the different stages of the wound repair process (Lucas et al., 2010).

There seems to be an association between macrophages and the response to injury in case when ciliary functions in tissues are damaged (Zimmerman et al., 2017). Data from primary ciliary dyskinesia (PCD), a genetic disorder in which mutations cause defects in all cilia, has shown that dysfunctional cilia reduce infection resistance and that the inflammatory response is aggravated by PCD monocytes (Cockx et al., 2017).

### 3.2. Mast Cells

Mast cells are multifunctional and highly granulated immune cells of the myeloid lineage that synthesize and store various mediators of inflammation (Krystel-Whittemore et al., 2016). They are located in mucosal and epithelial tissues throughout the body, specifically in areas below the epithelium, surrounding blood cells, SM, mucous, and hair follicles (da Silva et al., 2014; Krystel-Whittemore et al., 2016). Mast cells in the skin are activated by antigens through IgE binding and produce histamine, which is an essential chemical mediator of inflammation, among other bioactive substances (Kawamoto and Masuko, 2013).

There is increasing interest in mast cells due to their potential role in wound healing (Trautmann et al., 2000; Masatoshi et al., 2002; Chisholm and Greene, 2011). Histamine stimulates the proliferation of fibroblasts derived from both normal skin and keloid and scar tissue (Russell et al., 1977). Furthermore, the proinflammatory cytokine TNF-α is produced by mast cells (Walsh et al., 1991). It has been shown that TNF-α induces fibroblast proliferation and collagen metabolism, and it also increases the length of PC. This may play a critical role in wound healing (Tharp, 1989).

### 3.3. T Cells

T cells or T lymphocytes are pivotal leukocytes in the immune system and regulators of inflammation (Eagar and Miller, 2019). Circulating naïve T cells are activated when they encounter their cognate antigens, leading to cell proliferation and differentiation into effector T cells (Takamura, 2018). It has been shown that T cell activation is induced in the context of peptide-major histocompatibility complex ligands at the surface of antigen-presenting cells (APCs) via the T cell receptor. The immunological synapse (IS) is a highly specialized interface that forms between a T cell and an APC (Grakoui et al., 1999).

T cells are known to lack PC. Despite this fact, T cells possess certain cilia-like functions at the IS, a cell-cell junction between T cells and antigen-presenting cells, and they continue to express the proteins involved in ciliogenesis and use these proteins to build the IS (Cassioli and Baldari, 2019). There is increasing interest in the role of the IS since T cells may express several common proteins that are present in both the IS and PC. Although the IS and PC are structurally different, many ciliary proteins are recognized as active participants in IS-related functions in non-ciliated T cells (Cassioli and Baldari, 2019). The IS and PC still have limited structural and functional similarities, for instance, providing sensing and signaling platforms in cells.

However, PC are not generally found in hematopoietic cells (Cassioli and Baldari, 2019). Cells of hematopoietic origin still maintain some functions of PC, such as the activity of certain signaling pathways (Finetti et al., 2011).

### 3.4. Langerhans Cells

Langerhans cells (specialized dendritic cells, DCs), and CD8+ cytotoxic T cells (Maibach and Honari, 2014) reside in the skin's superficial epidermal layer (Clayton et al., 2017). There are also various specialized immune cells in the dermis, such as antigen-presenting dermal DCs, T cells, B cells, NK cells, mast cells, monocytes, and macrophages (Pasparakis et al., 2014).

Langerhans cells are bone-marrow-derived antigen-presenting immune cells located in the basal and suprabasal layer of the epidermis (Lombardi et al., 1993). Langerhans cells have characteristic features of dendritic cells, and the presence of PC in both Langerhans and dendritic cells has been shown (Toriyama et al., 2020). Granulocyte macrophage-colony stimulating factor (GM-CSF) is a hematopoietic growth factor that is mainly produced by immature Langerhans cells and stimulates the formation of PC in granulocytes and macrophages, activating the differentiation of granulocytes and macrophages from bone marrow precursor cells. Furthermore, PDGFRα localizes to PC, and PDGFα signaling promotes the proliferation of dendritic cells (Toriyama et al., 2020).

Interestingly, in the inflamed epidermis of atopic dermatitis patients, which has reduced barrier function, the number of cells with PC was increased, and atypically ciliated Langerhans cells and keratinocytes were found (Toriyama et al., 2020). As Langerhans cells present antigens to lymphocytes similar to macrophages, PC that regulate their activity are essential for the immune functions of the skin (Marks and Miller, 2013).

## 4. Primary Cilia and Neuronal Regulation of Cutaneous Immunity and Inflammation

It has been suggested that mechanical stress, which involves skin stretching, may result in the formation or aggravation of various cutaneous inflammatory disorders including pathological scars, atopic dermatitis, and psoriasis via stimulation of mechanosensitive nociceptors on sensory nerves in the skin (Akaishi et al., 2008; Choi and Di Nardo, 2018; Malakou et al., 2018). These sensory nerves release neuropeptides, such as substance P (SP) and calcitonin gene-related peptide (CGRP). As the nerve endings have contacts with virtually all epidermal and dermal cell types (Scholzen et al., 1998), the signals emanating from these nerves cause the activation of endothelial and vascular smooth muscle cell (VSMCs), leading to vasodilation and permeabilization of vessels (Akaishi et al., 2008). In addition, histamine release from mast cells is induced by SP (Columbo et al., 1996), which also further contributes to vasodilation and the permeabilization of vessels. These reactions are considered to result in neurogenic inflammation, in which central stimulation of sensory nerves evokes antidromic impulses that induce vasodilation and plasma extravasation and result in a local inflammatory response (Holzer, 1998; Brookoff, 2009), which contributes to the formation or aggravation of pathological scars, atopic dermatitis, and psoriasis (Akaishi et al., 2008). Furthermore, SP causes the production of TNF-α and IL-6 in mast cells (Azzolina et al., 2003), and these proinflammatory factors are associated with the appearance of pathological scars. Increased levels of SP stimulate the release of various other proinflammatory cytokines such as TNF-α, IFN-γ in the case of atopic dermatitis, and psoriasis (Remröd et al., 2007; Choi and Di Nardo, 2018). Neuropeptides and numerous cells also stimulate TGF-β, whose signaling pathway is coordinated by PC, and nerve growth factor (NGF) (Akaishi et al., 2008). NGF is thought to be one of the mediators of mechanical tension signals in hypertrophic scarring (Xiao et al., 2013). The expression of NGF is increased in the skin of patients with atopic dermatitis, and this seems to contribute to aggravation of the disease (Dou et al., 2006).

It has been observed that sensory neurons are associated with wound repair, and depletion of cutaneous sensory innervation is related to a delay of the wound healing process (Maggi et al., 1987; Carr et al., 1993; Smith and Liu, 2002; Barker et al., 2006). Further, sensory nerves seem to control physiological and pathological processes in the skin through the activation of target cells, which express receptors for neuromediators (Kruger, 1996; Roosterman et al., 2006). Thus, dorsal root ganglia or sensory neurons may not only receive and transduce mechanical and noxious stimuli but also play a valuable role in wound repair. However, the exact mechanisms are not yet known. Interestingly, exogenous administration of CGRP and SP is thought to promote wound closure (Engin, 1998; Delgado et al., 2005; Toda et al., 2008; Rook et al., 2009), and CGRP in particular has been observed to improve epithelial proliferation in some studies (Seike et al., 2002; Yu et al., 2009).

## 5. Primary Cilia, Granulation Tissue Formation, and Wound Healing

### 5.1. Endothelial Cells

In endothelial cells, PC are essential for sensing of blood flow shear stress (Chen et al., 2017). They also contribute to sensing of pH and oxygen (Anvarian et al., 2019).

Endothelial cell responses to FSS through PC help maintain barrier function, blood vessel wall permeability, and vascular tone (Jones et al., 2012; Peng et al., 2019). PC are also involved in endothelial cell inflammatory signaling, as their loss promotes vascular inflammation (Dinsmore and Reiter, 2016), and their length is controlled by cytokine stimuli, as mentioned above (Dummer et al., 2018). Defects in endothelial PC cause disorders of blood fluid-induced responses and result in vascular dysfunctions, such as hypertension, aneurysm, and atherosclerosis (Pala et al., 2018).

The pro-and antiangiogenesis phases of the wound healing process require precise sequential orchestration, and if poorly controlled, they can promote fibrotic scar formation (DiPietro, 2016). Interestingly, Chen et al. (2017) demonstrated that FFS sensing and Notch signaling through endothelial cell PC are required for arterial maturation and recruitment of vascular mural cells/pericytes in zebrafish, suggesting that endothelial PC may play a crucial role in controlling angiogenesis during wound healing.

### 5.2. Vascular Smooth Muscle Cells

Although not directly in contact with luminal flow and shear stress in intact arteries, VSMCs, and adventitial fibroblasts can be exposed to transmural interstitial flow (Shi and Tarbell, 2011). PC respond to mechanical deflection and integrin activation with an increase in intracellular calcium concentrations (Lu et al., 2008). Mechanosensing in VSMCs not only induces changes in contraction and cell morphology but also leads to altered production of cytokines and other vasoactive and pro-/anti-inflammatory signaling molecules (Shi and Tarbell, 2011).

BBSome in vascular smooth muscle were recently demonstrated to contribute to the control of vascular function and stiffness (Reho et al., 2019). The deletion of a gene encoding a critical BBSome component, Bbs1, resulted in vascular dysfunction, increased contractility, and reduced relaxation (Reho et al., 2019).

Furthermore, in contrast to non-ciliated cells, ciliated VSMCs showed more efficient migration in wound repair (Lu et al., 2008). Lu et al. (2008) found out that ciliary redistribution following wounding is regulated through the ciliary integrin-ECM interaction, as ciliary resettlement was decreased after β1-integrin blockade. Since VSMCs and the ECM are essential for regulating vascular tissue homeostasis, differentiation, and wound repair, cilium-mediated functions may be pivotal for maintaining vascular functional integrity (Raines, 2000). As compression therapy (discussed below) is one of the most widely used methods to prevent or reduce the appearance of scars in wound patients (discussed below), the mechanosensing via PCs may have important implications in both pathogenesis and also the treatment of such scars.

### 5.3. Fibroblasts

Dermal fibroblasts are dominant components of the skin's dermal layer, which resides underneath the epidermis. Based on anatomical location and structural and functional differences, these fibroblasts are divided into superficial (papillary) and deep dermal (reticular) fibroblasts (Brown and Krishnamurthy, 2018) as well as hair follicle-associated fibroblasts that reside in the immediate vicinity of hair follicles (Jahoda and Reynolds, 2001). However, these fibroblast populations only scratch the surface of fibroblast heterogeneity. Guerrero-Juarez et al. (2019) discovered at least 12 different fibroblast populations in murine skin wounds using single-cell resolution RNA sequencing.

Phenotypic differences between fibroblasts result in differences in ECM production and organization, growth factor production, and contributions to inflammatory responses (Doane and Birk, 1991). Fetal skin has been observed to heal scarlessly in mammals until a certain gestational age (Colwell et al., 2005). However, the exact mechanism underlying scarless fetal wound healing is not known, there are significant differences in the ECM, the inflammatory response, cellular mediators, gene expression, stem cell function, and fibroblast phenotypes between fetal and postnatal wounds (Shizuru et al., 2005; Larson et al., 2010). Collagen production is more tightly regulated in fetal fibroblasts (Gosiewska et al., 2001). A low level of TGF-β-1 has also been suggested to be associated with reduced scar formation (Shah et al., 1994).

PC regulate signaling pathways that are necessary for fibroblast migration during wound healing (Christensen et al., 2008). Signaling via PDGFRα regulates reorganization of the cytoskeleton and coordinates cell migration during wound healing (Schneider et al., 2010; Christensen et al., 2013). Moreover, fibroblast signaling through PDGFα has been shown to contribute to increased deposition of collagen and fibronectin, suggesting that PC signaling may guide dermal remodeling (Horikawa et al., 2015). PC in fibroblasts are increasingly recognized as a major regulator of ECM deposition in health and disease (Teves et al., 2019; Villalobos et al., 2019; Collins and Wann, 2020).

Although the frequency of PC on keloid or hypertrophic scar fibroblasts has been shown not to differ from that on normal fibroblasts (Strugnell et al., 1996), keloid fibroblasts show increased levels of PDGF receptors and demonstrate increased migration and proliferation in response to PDGFR ligand stimulation (Haisa et al., 1994). It is known that PC-associated PDGFR signaling activates the downstream JAK/STAT pathway, especially STAT1 and STAT3 (Vignais et al., 1996). Recently, Lee et al. (2019) implicated the STAT3 signaling pathway in keloid pathogenesis via RNA sequencing analysis of normal and keloid fibroblasts. Although these results indirectly suggest that fibroblast PC signaling could be involved in pathological scarring, direct experimental evidence to prove or disprove their role in this process is lacking.

Mechanical force applied to fibroblasts causes them to respond with signal transduction by the process of mechanotransduction. The mechanical force, or their stimuli, are then transduced into biochemical and gene expression signaling pathways. Thus, the stimuli alter the cellular function or induce apoptosis. Mechanical stimulation can also be converted into chemical signaling in cells such as fibroblasts, increasing fibroblast fibrotic gene expression, and increasing apoptosis. It has been suggested that cellular adhesions, which serve as mechanoreceptors in general, may also play an essential role in scar modulation after pressure therapy (Atiyeh et al., 2013).

### 5.4. Myofibroblasts

#### 5.4.1. Myofibroblasts and Wound Healing

In the maturation or remodeling phase of wound healing, increased mechanical tension and TGF-β signaling stimulate fibroblasts to differentiate into myofibroblasts, a highly contractile mesenchymal cell type. Myofibroblasts were initially described in granulation tissue during open wound healing as modulated fibroblasts present in all organs and numerous physiological conditions (Kalluri and Zeisberg, 2006; Hinz et al., 2012) or as modified fibroblasts that possessed characteristics of SM cells, such as bundles of microfilaments (Gabbiani et al., 1971). Myofibroblast phenotypes are modified by TGF-β, inflammatory factors, and immune cells, such as different macrophage phenotypes, ECM stiffness, and aging (Avery, 2017; Shook et al., 2018). Classically, myofibroblasts are identified by the expression of α-SMA, which promotes their contractility (Hinz et al., 2003). Myofibroblasts and their specific subpopulations can be identified by the expression of fibroblast activation protein alpha (FAPα), a membrane-bound serine protease, Pdgfra, stem cell antigen-1 (Sca1), integrin alpha-8 (Itga8), CD34, and dipeptidyl peptidase-4 (Dpp4, CD26) (Avery, 2017; Mah et al., 2017; Shook et al., 2018). Myofibroblasts with high FAP expression represent a reactive phenotype induced by low ECM stiffness and ECM fibronectin. These cells have been suggested to contribute to ECM synthesis and proteolysis, whereas SMA-expressing myofibroblasts exhibit a contractile phenotype induced by high ECM stiffness and type I collagen (Avery, 2017). The dynamic and multifaceted nature of myofibroblast phenotypes is further reflected by the term proto-myofibroblast, which was coined to describe an α-SMA-negative transitional phenotype that may also reflect (Tomasek et al., 2002; Hinz and Lagares, 2020) the reversibility of the myofibroblast phenotype (Tomasek et al., 2002; Nagaraju et al., 2019).

Myofibroblasts contribute to wound healing in various ways. They have the ability to secrete ECM molecules, such as collagen type I and collagen type III, and exert their contractile properties (Tomasek et al., 2002). The contractile apparatus of the myofibroblast comprises actin, myosin, and related proteins, such as alpha-smooth muscle actin (α-SMA), that are also found in other SM-expressing cells (Darby et al., 1990; Hinz et al., 2003; Van Caam et al., 2018). Myofibroblasts also form specialized adhesion structures with the ECM, which facilitate contraction of damaged tissue areas (Van Caam et al., 2018). Normally, during the resolution phase of tissue repair, myofibroblasts undergo apoptosis (Klingberg et al., 2013). Evasion of myofibroblast apoptosis, on the other hand, results in fibrosis and fibrotic disease (Hinz and Lagares, 2020). For instance, systemic sclerosis or scleroderma is an autoimmune disorder that typically results in fibrosis of the skin. Fibrosis is the hallmark of scleroderma and is described as excess deposition and accumulation of ECM in the dermis (Jinnin, 2010). Reduced apoptosis is evident, for example, in hypertrophic scars after burn injury (van der Veer et al., 2009; Liu et al., 2013).

It has been demonstrated that in such cases, collagen type I is replaced by collagen type III, which is typically present in remodeling tissues but not in normally healing wounds (Gabbiani et al., 1976). Therefore, the formation of granulation tissue accompanies the modulation of fibroblasts toward myofibroblasts during wound healing (Gabbiani, 2003). The progression from granulation tissue to scar tissue is known to involve the disappearance of myofibroblasts (Desmouliere et al., 1995). Hypertrophic scars or keloids appear when granulation tissue cells are not removed (Desmouliere et al., 1995) (see below). Further, most myofibroblasts express SM proteins, e.g., α-SMA and desmin. In general, α-SMA-expressing myofibroblasts are absent in keloids and normal scars but present in hypertrophic scars (Koese and Waseem, 2008).

#### 5.4.2. Myofibroblast Transformation

TGF-β signaling progresses through the formation of a heterotetrameric receptor confirmed to include type I and II TGF-β receptors (Huang and Chen, 2012). The assembly and activation of the receptor complex upon ligand binding results in phosphorylation and activation of the SMAD transcription factors SMAD2/3 (Huang and Chen, 2012; Clement et al., 2013).

Epithelial-myofibroblast transition (EMyT) is a unique and advanced type of Epithelial-to-mesenchymal transition (EMT), in which epithelial cells transdifferentiate into myofibroblasts that synthesize ECM proteins and produce contractile fibers (Radisky et al., 2007). TGF-β is known to be an inducer of EMT and EMyT (Xu et al., 2009). On the other hand, some studies have shown that the activation of TGF-β signaling alone is not sufficient to stimulate EMyT (Masszi et al., 2004; Sahin and Gungor, 2008). Moreover, it has also been suggested that a specific defect in PC activates EMT without TGF-β and exacerbates TGF-β-induced EMT (Han et al., 2018). Rozycki et al. indicated that the formation of the myofibroblast phenotype was followed by the absence of PC, regardless of whether the precursors were epithelial or mesenchymal cells. Further, the transition to the myofibroblast state is essential for deciliation because TGF-β is able to induce myofibroblast transition in mesenchymal cells (Rozycki et al., 2014).

Interestingly, PC have been demonstrated to regulate myofibroblast transition (Egorova et al., 2011). Rozycki et al. (2014) showed that PC of fibroblasts or epithelial cells are initially required for myofibroblast transition and growth but are subsequently lost upon acquisition of the myofibroblast phenotype. The EMyT and the fibroblast-to-myofibroblast transition are controlled by TGF-β signaling (Cigna et al., 2012). Regulation of PC signaling, assembly, or disassembly may thus have important therapeutic implications for pathologies that involve dysregulation of myofibroblast differentiation or persistence.

Myofibroblasts undergo apoptosis (Gabbiani, 1996), which is associated with TGF-β signaling (Zhang and Phan, 1999). TGF-β stimulates the synthesis of α-SMA and activates the production of collagen type I, which suggests that TGF-β regulates the tissue shape via modulation of ECM formation. TGF-β also initiates EMT and EMyT and thereby contributes to the regulation of wound healing. Moreover, PC undergo major alterations during EMyT (Rozycki et al., 2014). Amendt et al. (2002) showed that the expression of the dominant-negative type II TGF-β receptor in keratinocytes causes increased keratinocyte proliferation in the wound, which leads to strongly accelerated re-epithelialization.

These findings support the notion that TGF-β reduces re-epithelialization by repressing keratinocyte proliferation. Hence, reducing TGF-β signaling may contribute to better wound healing. It has also been suggested that the absence of PC causes dysregulated cell displacement during wound healing, leading to a reduced rate of wound healing and defects in wound closure (Schneider et al., 2010). Thus, PC are indispensable for wound healing and the formation of granulation tissue.

### 5.5. Keratinocytes

Keratinocytes are the major cell type of the skin's outer layer, the epidermis (Lee et al., 2013). The programmed process of keratinocyte renewal and differentiation generates and maintains the multilayered structure of the epidermis (Pincelli and Marconi, 2010). The epidermis spans from the self-renewing stem cell compartments to the cornified outermost layers and constitutes the protective barrier of the body (Simpson et al., 2011; Lu et al., 2015a). Epidermal stem cells reside in the basal layer of the interfollicular epidermis and in skin appendages (Evans and Kaufman, 1981; Molofsky et al., 2004; Cichorek et al., 2013). These cells are essential for wound healing (Raja et al., 2007; Pastar et al., 2008) and skin regeneration after injury (Burgoyne et al., 2009; Dehkordi et al., 2019).

The formation of PC on keratinocytes is linked to differentiation (Croyle and Lehman, 2011), whereas the disruption or loss of PC leads to keratinocyte hyperproliferation (Ezratty et al., 2011). Because the balance between keratinocyte proliferation and differentiation governs epidermal homeostasis, PC-associated signaling can greatly influence keratinocyte fate decisions and thus maintenance of the epidermal structure (Choi et al., 2016).

### 5.6. Bone Marrow-Derived Cells

Mesenchymal stromal cells (MSC) originate from the bone marrow are able to differentiate into various mesenchymal cell lineages; they are widely used in cell-based therapeutic approaches, including regeneration of damaged connective tissue (Ozawa et al., 2008). PC are present on MSCs. Interestingly, tumor necrosis factor-α (TNF-α) seems to activate the dose-dependent depletion of PC in MSCs, which suggests that PC can be a biological marker for the tumor-supporting properties of MSCs (Vézina et al., 2014).

### 5.7. Adipose Cells

Adipocyte precursor cells are activated and populate the wound during the proliferative phase of acute wound healing (Schmidt and Horsley, 2013; Guerrero-Juarez et al., 2019). Macrophages can contribute to the recruitment of these cells to the wound (Guerrero-Juarez et al., 2019). Adipocyte precursors, in turn, contribute to the recruitment of fibroblasts to the wound and promote dermal regeneration and remodeling (Schmidt and Horsley, 2013).

Schmidt and Horsley (2013) speculate that the activation of adipocyte precursors is driven by the interplay between immune cells and that intercellular signaling between fibroblasts and adipocytes could be mediated by PDGF and BMP signaling. Since these signaling pathways are governed by PC, these results further suggest a central role of signaling through preadipocyte and fibroblast PC in the control of wound healing. Dysregulated intercellular communication among adipocytes, immune cells, and fibroblasts may thus contribute to scarring through uncoordinated stimuli that regulate cell proliferation or ECM deposition.

The obesity phenotype of ciliopathies may be associated with these pathways since PC are related to Hh and Wnt signaling (Sen et al., 2009). BBS is a rare ciliopathy characterized by obesity, retinal dystrophy, postaxial polydactyly, and renal dysfunction (Forsythe and Beales, 2013). A dysfunctional BBSome protein complex controlling IFT transport of cargo, for example, receptor proteins, to and from the PC (Wingfield et al., 2018) can cause defects in ciliary signaling pathways, such as Wnt and Hh, that contribute to dysfunctional differentiation of adipocytes (Liu and Lechtreck, 2018). In preadipocytes, the BBSome is required for ciliogenesis (Sen et al., 2009).

## 6. Other Skin Cells With Less Established roles in Wound Healing and Scarring

### 6.1. B Cells

B cells, also called B lymphocytes, are a subclass of leukocytes that play an essential role in pathogen-specific immunity by producing antibodies (Stollar, 1998). Furthermore, they can also act as antigen-presenting cells (Rodríguez-Pinto, 2005). There has been increasing interest in B cells due to their potential role in the cutaneous immune system. *In vivo* and clinical studies have demonstrated that B cells have proinflammatory and suppressive roles in inflammatory skin disorders (Egbuniwe et al., 2015).

The IS is associated with B cell activation, as B cells can be activated by APCs, and B cell receptor signaling starts to form the IS when B cells contact membrane-bound antigens (Carrasco et al., 2004; Huang et al., 2005). The IS also works as a platform for B cells to recognize pathogenic antigens on APCs, similar to the T cell IS (Huang et al., 2005). Under certain conditions, immortalized T and B cells may form an initial primary cilium. However, PC do not exist in normal mature hematopoietic cells *in vivo*. Hence, in these cells, the expression of PC-related proteins may be severely reduced or even absent (Cassioli and Baldari, 2019).

### 6.2. Melanocytes

In the human skin, neural crest-derived melanocytes are distributed throughout the basal layer of the epidermis (Barbieri et al., 2014). The ratio of melanocytes to keratinocytes varies from 1:4 to 1:10 depending on the location (Norris, 2012).

Extrinsic factors, such as ultraviolet radiation, the cellular microenvironment, hormones, and inflammation, modulate the degree of skin pigmentation (Costin and Hearing, 2007; Arndt et al., 2013). The skin pigment melanin is synthesized in melanosomes within melanocytes. Melanosomes are delivered to keratinocytes via the dendrites of melanocytes (melanocyte-to-keratinocyte transfer). Melanin protects the inner microenvironment of keratinocytes, including their DNA, from UV radiation-induced damage (Wu and Hammer, 2000; Bowman and Marks, 2018). Genetic determinants regulate the quantity of melanin produced by melanocytes and the size of the melanosomes that are transferred to keratinocytes (Bessou-Touya et al., 1998). Although the association between skin pigmentation and PC remains unclear, PC are indeed found on melanocytes. However, they are frequently lost in melanomas (Kim et al., 2011; Le Coz et al., 2014). Sensing and signaling through PC may thus contribute to the functions of differentiated melanocytes.

### 6.3. Merkel Cells

Merkel cells are located in the basal layer of the epidermis, accounting for <5% of the total epidermal cell population (McGrath and Uitto, 2010). They are essential for neuroendocrine functions, and due to their synapse-like contacts with neurons, these cells are instrumental for sensing pain and mechanical stimuli such as light touch and hypotonia-induced cell swelling (Abraham and Mathew, 2019). The surface of Merkel cells is characterized by numerous microvilli that participate in sensory functions (Toyoshima et al., 1998), but the role of PC remains unclear. However, the PC-associated Hh signaling cascade guides cells to the skin sensory lineage (Xiao et al., 2016), contributing to the formation of the touch dome, a sensory organ comprised of Merkel cells and specialized keratinocytes.

## 7. Fibrosis, Keloids, and Hypertrophic Scars

Fibrosis is a pathological process that results from chronic inflammatory reactions caused by, for instance, tissue injury. Fibrosis is characterized by excess accumulation of ECM and can lead to permanent scarring e.g., keloids, and hypertrophic scars in the skin (Wynn, 2008). The inflammatory response is aggravated by increased accumulation, enhanced production, or failure to remove stimulating factors; if unregulated, inflammation can drive fibrosis (Wynn and Ramalingam, 2012). Moreover, fibrosis is known to evoke persistent myofibroblast activation and inflammation, which can cause severe organ dysfunction (Hinz and Lagares, 2020).

Keloids are pathological scars that are described as a benign fibroproliferative dysfunction characterized by abnormal excessive deposition of collagen during wound healing. Keloids extend beyond the borders of the original wound and invade the normal skin, which distinguishes these scars from other pathological hypertrophic scars (Lee et al., 2004). Keloids are common in African, Spanish, and Asian populations, with an incidence ranging from 4.5 to 16%, and cause itching, pain, and a burning sensation (Niessen et al., 1999). Keloids can occur within years, and more likely after an inciting stimulus such as dermal injury or an inflammatory process (Limandjaja et al., 2020); however, they are most often found on the chest, shoulders, upper back, and back of the neck, where skin tension is higher than in other areas of the body (Ogawa et al., 2012; Liu et al., 2020). In addition, keloids tend to occur on the earlobes as a result of ear piercing, burns, or surgical procedures. The earlobes consist of tissue with little to no tension, and Chike-Obi et al. (2009) suggested that keloids can also form in areas with minimal tension due to the proliferation of dermal elements after injury. Keloids do not regress, unlike hypertrophic scars (Murray, 1994), and the exact pathogenic mechanism of keloid formation is still unclear. Various mechanisms of healing disorders seem to be associated with keloid formation.

Ogawa (2017) suggested that keloids and hypertrophic scars are derived from an abnormal inflammatory reaction in the skin since proinflammatory factors, including IL-1α, IL-1β, IL-6, and TNF-α, are associated with keloid formation. Additionally, disorders of vascular cells can cause pathological scars since inflammation stimulates excessive angiogenesis, endothelial disorder, and vascular hyperpermeability (Huang and Ogawa, 2020). It has been proposed that mechanical tension promotes the dysregulation of cell proliferation and apoptosis during wound healing and may result in keloids and hypertrophic scars (Pozos, 2014; Harn et al., 2019; Ogawa, 2019).

Myofibroblasts create specialized adhesion structures with the ECM, which causes tension in their surroundings and contracts damaged tissue. Generally, expansion of the ECM is caused by increased skin tension, resulting in stiffer tissue. Harn et al. (2019) suggested that keloids form differently than hypertrophic scars and other normal scars because of the variation in scar sensitivity to skin tension. PC and the ECM are functionally closely connected, and defects in PC are associated with deregulated fibrosis. Thus, we hypothesize that defects in PC may facilitate not only the development of ciliopathies (Seeger-Nukpezah and Golemis, 2012) but also the formation of keloids. Moreover, keloids contain remarkably increased and disorganized collagen bundles. Fibroblasts in keloids respond abnormally to growth factors and stimuli arising from the ECM during wound healing, and the delay of fibroblast apoptosis in keloids seems to be associated with uncontrolled production of excessive amounts of collagen (Huang et al., 2013).

Hypertrophic scars can occur within 4–8 weeks after injury and occur in up to 90% of burn patients (Oosterwijk et al., 2017). Hypertrophic scars are more common than keloids. Hypertrophic scars are a fibrotic disorder resulting from unchecked proliferation of fibrous tissue following injury to the skin. They are described as raised, erythematous, itchy lesions (Peacock, 1970). Unlike keloids, hypertrophic scars ultimately regress and occur mainly around joint areas- the elbows and knees (Niessen et al., 1999; Butzelaar et al., 2016). The recurrence rates of hypertrophic scars are lower than those of keloids after excision (Gauglitz et al., 2011). Hypertrophic scars and keloids have excessive collagen content. While hypertrophic scars contain well-organized type III collagen positioned in parallel to the epidermal surface and abundant myofibroblasts, keloids contain disorganized type I and III collagen with no myofibroblasts (Slemp and Kirschner, 2006). Both scar types show overexpression of numerous fibroblast-specific proteins, such as fibronectin (Sephel and Woodward, 2001), which may sustain pathological wound healing or promote downregulation of the mechanisms that terminate the wound healing process.

## 8. Primary Cilia-Targeted Anti-Fibrotic Therapeutic Approaches

The intricate mechanisms involved in PC assembly and disassembly, as well as the molecules and pathways involved in their regulation, constitute some of the many possible targets for therapeutic interventions that involve signaling through the PC. Examples of such targets include suppressors of PC formation (mitostatin, NDEL1, CP110 KIF24, and INPP5E), facilitators of PC resorption (AURKA, HDAC6, CDC20, and DYNLT1) and promoters of ciliogenesis (MST1/2, the CRL3-KCTD17 complex, PLK4, NPHP6, and Rab8a) (Walz, 2017).

Another level of regulation could involve proteins and pathways associated with PC function. These functional regulators include IFT proteins of, for example, the BBSome that transport cargo to and from PC, kinesins, and dyneins as well as the IFT-A and -B complexes (Ishikawa and Marshall, 2011; Basten and Giles, 2013).

Corticosteroids are the first-line clinical treatment of keloids and hypertrophic scars. Fibroblast production and proliferation capabilities are affected by corticosteroids. They inhibit fibroblast growth and cause fibroblast degeneration, and have been shown to affect the length of PC (Khan et al., 2016). Corticosteroids seem to also induce an increase in the production of basic fibroblast growth factor and additionally a decrease in the observed production of TGF-β1 by human dermal fibroblasts, endogenous VEGF, and IGF-1 (Roques and Téot, 2008). As glucocorticoids act as general suppressors of inflammation the role of ciliary signaling modulation in scar suppression is not yet clear.

In addition to corticosteroids, many other drugs and small molecules have also been shown to affect the length of cilium (Khan et al., 2016). These include compounds that increase cyclic AMP, such as phosphodiesterase inhibitors or activators of cAMP production, for example, forskolin (Miyoshi et al., 2011) or cholera toxin (Hirst, 2001). Moreover, among environmental factors, hypoxia exerts cell type-dependent regulatory effects on ciliogenesis (Miyoshi et al., 2011), possibly through pVHL signaling (Thoma et al., 2007).

Therapeutic approaches to induce myofibroblast apoptosis or sensitize myofibroblasts to apoptosis are currently under intensive investigation, and several drugs are undergoing phase II/III studies for the treatment of fibrotic diseases (Hinz, 2020). Recently, Choi et al. (2019) demonstrated that PC can protect neuronal cells from apoptosis and that treatment with ciliobrevin D, a drug that inhibits dynein function and disrupts the formation of PC, sensitizes these cells to apoptosis. Their results indicating that the pharmacological modification of PC affects cellular programmed cell death pathways suggest that PC may serve as an attractive target to induce myofibroblast apoptosis for the treatment of fibrosis and pathological scars.

As PC have prominent roles in a number of intracellular pathways several potential approaches to suppress pathological sar formation that targets these pathways can be envisaged. One possibility is to interfere with or inhibit myofibroblast transdifferentiation via different PC-associated signaling pathways, such as TGF-β regulated by a CTGF-dependent pathway in concert with either EGF or IGF-2 (Grotendorst et al., 2004). Moreover, ligands of the Wnt/beta-catenin-pathway can upregulate TGF-β signaling and induce myofibroblast differentiation.

Blocking the canonical Wnt receptor (β-Catenin signaling route) or interfering with this signaling pathway through PC could offer a target to decrease myofibroblast transformation.

The role of PC in mechanotransduction and the associated downstream signaling pathways, including TGF-β/Smad, mitogen-activated protein kinase (MEK), RhoA/ROCK, Wnt, and TNF-α could also act as therapeutic targets.

It has been suggested that disruptions in cellular mechanotransduction can cause impaired wound healing and scar formation (Jaalouk and Lammerding, 2009). Mechanotransduction is also one of the mechanisms through which Negative Pressure Wound Therapy (NPWT), also called vacuum-assisted closure (VAC) exerts its therapeutic effects in wound treatment (Huang et al., 2014). Further, since PC length is thought to be critical to cellular mechanotransduction (Spasic and Jacobs, 2017), treatments that modify the length of PC could possibly be utilized to enhance the effects of NPWT.

Increasing mechanical tension is key to the development of scar tissues through biochemical signals (Barnes et al., 2018), and it is possible to reduce scar formation by modifying these signals, including neutralization of TGF-β or addition of TGF-β3 (Shah et al., 1995). There are more techniques to reduce mechanical forces/tension to treat pathological scars, such as silicone gel sheets and paper tape. Silicone gel sheets reduce tensile stresses in the wound (Akaishi et al., 2010). Moreover, they are thought to decrease TGF-β and TGF-β2 expression in fibroblasts (Kuhn et al., 2001). Paper tape also seems to reduce scarring by reducing wound tension (Rosengren et al., 2013). Clinically, there are reports that paper tape reduces scar volume significantly and reduces mechanical tension, which results in minimal scar formation (Atkinson et al., 2005; Rosengren et al., 2013). A meta-analysis also indicated that pressure therapy was effective for hypertrophic scar patients, improving pigmentation, and redness and even reducing scar coloration (Ai et al., 2017).

Pressure therapy, also known us compression therapy represents the standard care for preventing and treating hypertrophic scars after burn injury. For example, elastic bandages or compression garments are used as compression therapy. It has been suggested that pressure may affect directly cellular scar components (Li-Tsang et al., 2010). There seems to be an increase in ECM rigidity produced by compression garments leads to a higher level of mechanoreceptor activity and increased cellular apoptosis (Atiyeh et al., 2013). Given the mechanosensory functions of PC, it seems possible that they may contribute to these effects of pressure therapy.

## 9. Conclusions

Our understanding of the role of PC in regulating the activity of a number of signaling pathways has improved considerably due to the growing interest in PC. Here, we reviewed the roles of different key PC signaling pathways and the related cells and discussed their possible impacts on scar formation and wound healing. As PC have central roles in many pathways that regulate the cutaneous wound healing process, their dysfunction is hypothesized to cause aberrant wound healing and repair resulting in the appearance of pathological scars. However, this hypothesis needs further study. Understanding and exploring this mechanism may help to promote new breakthroughs and the development of effective medical solutions that can alleviate pathological scars.

## Author Contributions

MH and EK drafted the paper. RO, VJ, HL, and JV reviewed and edited the manuscript. All authors read and approved the final manuscript.

## Conflict of Interest

The authors declare that the research was conducted in the absence of any commercial or financial relationships that could be construed as a potential conflict of interest.

## Abbreviations

BBS: Bardet-Biedl syndrome
BMP: bone morphogenetic protein
ECM: extracellular matrix
EMT: epithelial-to-mesenchymal transition
EMyT: epithelial-myofibroblast transition
GPCRs: G-protein-coupled receptors
Hh: Hedgehog
IFT: intraflagellar transport
IGF-1: insulin-like growth factor 1
IL: Interleukin
IS: the immunological synapse
MSC: mesenchymal stromal cells
PC: primary cilia
PDGF: platelet-derived growth factor
RTKs: receptor tyrosine kinases
SM: smooth muscle
SMAD: small mothers against decapentaplegic
TGF-β: transforming growth factor-β
TNF-α: tumor necrosis factor-α
VSMCs: vascular smooth muscle cells.
